# Impact of Life Experiences and Use of Web 2.0 Tools in Adults and Older Adults

**DOI:** 10.3389/fpsyg.2019.02158

**Published:** 2019-09-27

**Authors:** Cristina Díaz-Prieto, Jesús-Nicasio García-Sánchez, Alejandro Canedo-García

**Affiliations:** ^1^Personality, Evaluation and Psychological Treatment, Department of Psychology, Sociology and Philosophy, Universidad de León, León, Spain; ^2^Developmental and Educational Psychology, Department of Psychology, Sociology and Philosophy, Universidad de León, León, Spain; ^3^Educational Psychology, Department of Humanities, Universidad de la Costa, Barranquilla, Colombia

**Keywords:** differential patterns, life experiences, quality of life, practices, psychological profiles, web 2.0 tools

## Abstract

The aim of this study was to explore the relationship between favorable and stressful life experiences and perceived quality of life, practices that promote quality of life, psychological profiles, and the daily use of web 2.0 tools in adults and older adults. An online questionnaire was designed, which was administered to 1,095 Spanish adults and older adults, and conducted descriptive and multivariate analyses using the general linear model. Our results showed that favorable and stressful life experiences alike were associated with differential patterns in psychological profiles, perceived quality of life and daily activities and practices that affect quality of life, including the use of web 2.0 tools. Favorable life experiences mainly affected psychological profiles and the use of web 2.0 tools, whereas stressful life experiences affected the other factors analyzed. Statistically significant differences were not found according to age and gender. These findings have important implications for promoting successful psychological and social interventions.

## Introduction

As population aging becomes one of the main challenges for societies worldwide, there is a growing interest in addressing the determinants that have an impact on the well-being and quality of life of the elderly, among them, the life experiences. People's life experiences largely determine who they become. Their psychological profiles, personalities, lifestyles, moods, routines, interests, and even identities are partially shaped by the events that have happened in their lives, including the drastic changes that society has witnessed in recent years driven by the irruption of new technologies (Bai et al., 2014; El Haj and Antoine, 2017). Without a doubt, this change has largely been defined by the establishment of the internet and web 2.0 tools as an integral part of our lives and social participation, tools that allow to create and share information, collaborate, promote social contacts, and full integration into society. These technologies have been changing over the years. The main contribution of web 2.0 is the user's participation in the environment, creating and sharing information, unlike what happened in the web 1.0. Taking this into account, web 2.0 could be defined as an environment that favors the communication, collaboration, social relations, exchange, creation, collection, and transformation of content. Web 2.0 technologies include a wide range of tools, including blogs, social networks, wikis, podcasts, among others (Açikgül and Serdar, 2019). This has marked a before and after in the way in which we handle information, interact with each other and with the world around us and carry out our daily activities (Díaz-Prieto and García-Sánchez, 2016). Although data are available on the impact of life experiences on psychological profiles, practices and routines, and more generally on life satisfaction, well-being and quality of life in adults and older adults (Thomsen et al., 2016) little is known to date about the relationship between life experiences and the use of web 2.0 tools.

Studies on life experiences have traditionally employed techniques such as reminiscence therapy, a review of life or significant life events, reminiscence writing and life stories, with varying results. While some studies have highlighted the benefits, others have called these into question and have identified potential difficulties. Those that have found benefits include studies suggesting that these interventions exert a positive effect on depressive symptoms, anxiety, loneliness, socialization, memory, and self-esteem, and more generally on physical and mental health, life satisfaction, well-being, and quality of life, in both the healthy population and the population affected by a range of psychological, cognitive, affective-emotional, behavioral, or social problems (Hyams and Scogin, 2015; Latorre et al., 2015; Lopes et al., 2016; El Haj and Antoine, 2017; Wren, 2017). However, other studies have indicated the maladaptive effect of reminiscence, whereby experiences may be emphasized in an unhealthy manner or prompt rumination, self-blame and pessimism, and memories of these may undermine happiness, satisfaction, and well-being or trigger a series of negative emotions (Henkel et al., 2016; Stikkelbroek et al., 2016). Moreover, most studies have focused on analyzing stressful life events, their negative consequences and the coping strategies employed to get over them, especially in the older population (Latorre et al., 2015; Randall et al., 2015; Lasgaard et al., 2016). These studies have identified a series of events that negatively affect mental health, well-being and quality of life, including illness, grief, loneliness, social problems, changes of residence and work or financial problems, among many others (Donoghue et al., 2016; Chukwuorji et al., 2017; Tveit-Sekse et al., 2019). For example, theoretical approaches to solitude have demonstrated that important life events such as the death of a loved one or divorce prompt changes in interpersonal relations, triggering or perpetuating feelings of loneliness (Lasgaard et al., 2016). Other studies have focused on analyzing the psychopathological effect of stressful life events, associating them with high rates of anxiety and depression (Eisenbarth et al., 2019) and changes in personality traits (Bleidorn et al., 2018). Some studies have found that the negative life events accumulated throughout the life cycle have long-term effects on well-being. Thus, negative socio-economic circumstances, emotional abuse and neglect during childhood and negative socio-economic circumstances, sexual abuse, emotional abuse and neglect, relational stress, and problem behavior during late adulthood, are associated with higher rates of depression in old age (Kraaij and Wilde, 2001). Life experiences thus have a proven impact on psychosocial and emotional profiles and perceived quality of life, although this evidence is mainly based on negative rather than favorable life experiences and their consequences. It therefore seems pertinent to analyse the extent to which favorable life experiences are related to psychological profiles, given their plausible impact on the well-being and quality of life of adults and older adults.

Just as life experiences influence quality of life, it has also been found that various empirical evidence-based practices contribute to its optimization. Among others, physical exercise, mental activity, and self health care have proven effective in enhancing quality of life and promoting active aging (Foster and Walker, 2015; Hongthong et al., 2015; Kim et al., 2015; Marcus-Varwijk et al., 2016). Some studies have found how certain activities and practices promote the quality of life of adults and seniors: have good social relationships, help and support; living in a nice home and neighborhood, safety, use of community facilities and services; participate in leisure activities; social participation; have a positive psychological perspective and acceptance of circumstances that can not be changed; good health and mobility; and have enough money to satisfy basic needs, enjoy life and preserve independence and control of life (Gabriel and Bowling, 2004). However, the data on how life experiences determine such practices are scant.

Studies have also been conducted on the relationship between life experiences and pathological, but not day-to-day, internet use. An abundance of research exists on the predictors, patterns, and benefits of use of this medium in the adult population (Zheng et al., 2015; Díaz-Prieto and García-Sánchez, 2016; Marston et al., 2016); however, few studies have analyzed the impact of life experiences on use of the internet and web 2.0 tools, and those which have done so, have analyzed stressful life events and their impact on problematic internet use, since this medium often serves as a coping, refuge or escape mechanism, being used as a strategy to achieve mental disconnection or seek information and support (Li et al., 2010; Chan, 2015; van Ingen et al., 2016; van Ingen and Matzat, 2018; Xiao et al., 2019). According to these studies, internet addiction is a response to stressful life events that generate psychological stress in individuals who use this medium as a way of coping and regulating negative emotions in the absence of other types of positive coping strategies (Salovaara et al., 2010; Li et al., 2016). Other studies have used the internet as a tool to implement interventions based on a review of life, finding positive effects on depression, well-being, self-esteem, and obsessive reminiscence (Preschl et al., 2012). Nevertheless, further research is required to analyse the relationship between favorable as well as stressful life experiences and the use and benefits of web 2.0 tools.

Consequently, the research question that guided the present study was whether a relationship existed between favorable and stressful life experiences and perceived quality of life, practices that promote quality of life, psychological profiles and the daily use of web 2.0 tools in adults and older adults. Given that aging involves a wide range of life experiences, it would be expected to find differential patterns in quality of life practices, perceived quality of life, psychological profiles, and daily use of web 2.0 tools according to subjects' favorable and stressful life experiences, age, and gender.

## Methods

### Participants

An online assessment instrument was administered to 1,095 Spanish adults and older adults, 439 of whom were men and 656 women (Table 1). The sample was recruited through various institutions, organizations, centers, public and private universities, and university programmes for older adults throughout Spain, from September 2016 to February 2017. The inclusion criteria were: (i) people aged over 18 years old; (ii) informed consent to participate; (iii) basic digital competence; and (iv) sufficient autonomy to answer assessment instruments themselves.

**Table 1:** Distribution of participants by age and sex (*n* = 1,095).

	** <55**		**55–60**		**61–65**		**66–70**		**>70**		**Total**
	***N***	**${\bar{\text{X}}}_{age}$**	***N***	**${\bar{\text{X}}}_{age}$**	***N***	**${\bar{\text{X}}}_{age}$**	***N***	**${\bar{\text{X}}}_{age}$**	***N***	**${\bar{\text{X}}}_{age}$**	
Male	176	34	59	58	77	63	75	68	52	76	439
Females	322	33	79	58	100	63	88	68	67	75	656
Total	498		138		177		163		119		1,095

### Instrument and Variables

The instrument *Practices in Adults and Older Adults* (Spanish acronym: PRAMA) was designed using the *Google Forms* tool, and administered it online. This instrument consists of six scales which measure the following specific variables:

1. PRAMA-DD: this includes a series of items related to sociodemographic data: *sex, age, marital status, place of origin, place of residence, educational level, employment status, occupation, economic level, indicator of independent living*, and *degree of independence*.
2. PRAMA-PQL: this includes 15 items that measure perceived quality of life in 15 areas of life, namely: *physical health, mood, memory, family, friends, intimate relationships, place of residence, ability to meet basic needs, ability to perform household tasks, ability to perform tasks outside the home, leisure and entertainment, money, occupation, personal satisfaction*, and *life in general*. The questionnaire asked about how the participants valued their life in different areas (For example: How do you rate your mood?)
3. PRAMA-PRA: this assesses the frequency of performing empirical evidence-based practices that promote quality of life, including: *physical exercise, mental activity, self-care activities, meetings and contact with relatives and friends, intimate relationships, training activities, leisure and social activities, tourist activities*, and *volunteering*. For example, How often do you practice physical exercise?
4. PRAMA-LE: this includes two subscales that measure favorable (LE-FAV) and stressful (LE-STR) life experiences. The subscales share a number of items in common that assess the following aspects: *area (Example: In relation to what area was that event), stage (Example: At what stage of your life did that event happen?), description of the most important life event from a small narrative, emotions (Example: What did you feel?), affect in the short and medium term (Example:* What effects did this event have in the short and medium term?), and *present influence (Example: Does that event influence your present life)*. In addition, LE-STR includes a question that evaluates the *coping strategies* employed, namely: *acceptance, denial, active, planning, self-distraction, emotional support, instrumental support, emotional discharge, resignation, self-criticism, positive reformulation, humor*, and *religion*.
5. PRAMA-psychological: this is based on the INMA-psychological scale (Díaz-Prieto and García-Sánchez, 2016), which has shown satisfactory validity and theoretical and construct reliability (Cronbach's α = 0.826). It assesses several psychosocial and emotional indicators, namely: *emotional intelligence (Example: Indicate how often you recognize your feelings), achievement motivation (Example: Indicate how often you persist until you achieve your goals), social dimension (Example: Please, indicate how much you agree with the following statements- I have a broad social circle)*, and *self-efficacy in active aging (Example: To what extent do you feel able to be autonomous and independent to manage your money?)*.
6. PRAMA-Internet: this is adapted from the INMA-Internet scale, and includes a series of items that assess the *use of web 2.0 tools* (Example: Indicate which one or which of the following computer or internet tools you use or have used: communication tools, social networks, email, image and sound tools, Apps, browsers and search engines, cloud tools, functional tools, educational tools, tools for selecting, classifying and sharing information, and office automation tools) and the *perceived benefits (Example: Since I use…I carry out activities for myself that I did not do before)*.

Taken together, the instrument showed acceptable psychometric properties, with satisfactory content, theoretical and construct validity, as well as reliability, obtaining a total Cronbach's alpha of 0.720. By individual scale, a Cronbach's alpha was obtained of 0.748 for the PRAMA-PQL scale, 0.819 for PRAMA-PRA, 0.641 for PRAMA-LE, 0.769 for PRAMA-psychological, and 0.742 PRAMA-Internet. The average variance extracted (AVE) are above 0.50 in the different scales. The composite reliability (CR) goes from 0.80. Proven factor analysis models, the determinants are <0.001, as well as Barlett's sphericity tests. The KMO contrasts give above 0.85–0.99.

### Design and Procedure

The six scales comprising the instrument were designed following a review of various national and international descriptive and intervention studies and the instruments used in relation to empirical evidence-based quality of life practices, perceived quality of life, life experiences, psychosocial and emotional variables and internet use. Having designed the instrument and selected the type of sample, a pilot study was conducted with two groups of participants in a university programme for older adults in León (Spain) and another group of people attending a training course on technology tools, in order to determine the time required to complete the questionnaire, detect problems related to item interpretation and identify other problems that might arise during questionnaire completion. Subsequently, potential participants were contacted in person, by telephone, fax and the internet, to inform them about the study objectives and request their participation. Prior to completing the questionnaire independently at the time and place of their choice, participants gave their informed consent in accordance with the ethical and professional conduct rules applicable to all scientific research. The maximum time required to complete the questionnaire was 30–35 min, although there were differences depending on the participants' level of digital competence. Once the questionnaires were completed, the results were extracted in Excel format and codified before conducting the pertinent statistical analyses.

### Statistical Analysis

First, descriptive analyses were conducted (frequencies and percentages, means, and standard deviations). Normal distribution of the variables was confirmed by calculating skewness and kurtosis. Multivariate analyses were performed based on general linear models (GLM), using the IBM statistical software package SPSS Statistics 24.0. And for the calculation of Macdonald's omega/composite reliability, and average variance extracted indexes, Excel spreadsheet programs were carried out through from the pattern matrices of the factorial analysis of the instruments.

## Results

Statistically significant results for both of the grouping variables considered, with large effect sizes were found: (i) favorable life experiences [λWilks = 0.061; *F*_(1, 536, 6, 926)_ = 1,886; *p* ≤ 0.001; η^2^ = 0.295] and (ii) stressful life experiences [λWilks = 0.005; *F*_(3, 281, 14, 119)_ = 1,576; *p* ≤ 0.001; η^2^ = 0.267]. Statistically significant differences according to age and gender were not found.

Similarly, the test for between-subject effects yielded statistically significant relationships, as will be described in four subsections that follow.

### Differential Patterns of Perceived Quality of Life and Psychological Profiles According to Favorable Life Experiences

Subjects' *favorable life experiences* were associated with differential patterns in their *perceived quality of life* and *psychological profiles* (Table 2). For example, people who highlighted a workplace-related event scored higher on occupation-related perceived quality of life (e.g., perceived quality of life-occupation, *M*_job_ = 3.05 vs. *M*_finances_ = 2.57, *p* = 0.01). Similarly, favorable finance-related events seemed to be related to greater achievement motivation (e.g., total MOTIVATION, *M*_finance_ = 11.71 vs. *M*_education_ = 10.01, *p* = 0.02).

**Table 2:** Differential patterns in perceived quality of life and psychological profiles according to favorable life experiences.

**Variables**	**Physical health**		**Mental health**		**Social**		**Finances**		**Job**		**Education**		**Legal**		**Important life change**		**Other**		***F***	***p***	**η^**2**^**
	**$\bar{\text{X}}$**	**σ**	**$\bar{\text{X}}$**	**σ**	**$\bar{\text{X}}$**	**σ**	**$\bar{\text{X}}$**	**σ**	**$\bar{\text{X}}$**	**σ**	**$\bar{\text{X}}$**	**σ**	**$\bar{\text{X}}$**	**σ**	**$\bar{\text{X}}$**	**σ**	**$\bar{\text{X}}$**	**σ**			
**PERCEIVED QUALITY OF LIFE**																					
Friends	3.09	0.62	3.02	0.64	3.21	0.65	2.71	0.73	3.07	0.73	3.13	0.67	2.01	0.01	3.17	0.71	3.26	0.62	2.29	0.02	0.02
Intimate partner	2.60	1.07	2.35	1.01	2.98	0.98	3.01	0.96	2.62	1.02	2.62	1.03	2.01	1.41	2.84	1.02	2.61	0.89	3.15	0.01	0.02
Occupation	2.73	0.89	2.58	0.88	2.87	0.81	2.57	0.94	3.05	0.77	2.68	0.88	1.01	0.01	2.95	0.87	2.65	0.98	3.68	0.01	0.03
**PSYCHOLOGICAL PROFILE**																					
Emotional intelligence—own emotions	4.02	0.64	4.17	0.67	4.36	0.50	4.26	0.64	4.06	0.61	3.01	0.01	4.33	0.63	4.13	0.55	4.02	0.64	3.35	0.01	0.03
Motivation—intrinsic	4.23	0.72	4.06	0.70	3.86	0.95	4.24	0.78	4.20	0.78	3.50	0.71	4.14	0.74	4.26	0.81	4.23	0.72	1.97	0.05	0.02
Motivation—extrinsic	3.30	1.10	3.18	0.93	2.64	1.15	3.30	1.14	3.12	1.10	3.01	0.01	3.21	0.95	2.87	1.14	3.30	1.10	1.98	0.05	0.02
Total motivation	11.70	1.51	11.36	1.48	10.29	1.20	11.71	1.58	11.47	1.59	10.01	1.41	11.51	1.47	11.52	1.76	11.70	1.51	2.32	0.02	0.02
Social dimension—emotional support	4.14	0.94	4.28	0.82	4.01	1.04	3.88	1.03	4.19	0.91	3.01	1.41	4.22	0.82	3.96	1.11	4.14	0.94	2.93	0.01	0.02
Social dimension—information support	4.14	0.86	4.01	0.92	3.50	0.86	3.66	1.06	3.89	0.91	3.01	1.41	4.05	0.84	4.09	0.90	4.14	0.86	2.55	0.01	0.02
Social dimension—subjective assessment	4.16	0.75	4.17	0.78	3.79	0.98	4.02	0.89	4.04	0.72	2.50	0.71	4.13	0.82	4.17	0.98	4.16	0.75	2.08	0.04	0.02
Total social dimension	39.12	4.84	39.18	5.37	36.21	4.74	37.88	6.17	38.48	5.66	29.01	4.24	39.21	5.19	39.52	5.61	39.12	4.84	1.98	0.05	0.02
Self-efficacy—physical exercise	4.35	0.87	4.54	0.75	4.14	0.86	4.43	0.90	4.25	0.92	2.01	0.01	4.68	0.68	4.52	0.67	4.35	0.87	5.31	0.01	0.04
Self-efficacy—mental activity	4.70	0.51	4.70	0.59	4.50	0.52	4.54	0.70	4.56	0.65	3.01	0.01	4.73	0.57	4.65	0.65	4.70	0.51	2.97	0.01	0.02
Self-efficacy—social relationships	4.42	0.82	4.63	0.67	4.50	0.86	4.59	0.69	4.39	0.89	3.50	0.71	4.71	0.60	4.44	0.73	4.42	0.82	2.77	0.01	0.02
Self-efficacy—leisure	4.51	0.67	4.70	0.57	4.71	0.47	4.65	0.66	4.43	0.78	3.50	0.71	4.73	0.54	4.48	0.95	4.51	0.67	3.89	0.01	0.03
Self-efficacy—management of finances	4.47	0.86	4.72	0.54	4.79	0.43	4.74	0.55	4.58	0.72	3.50	2.12	4.81	0.46	4.70	0.64	4.47	0.86	2.51	0.01	0.02
Self-efficacy—activities outside the home	4.56	0.83	4.76	0.51	4.50	0.65	4.71	0.59	4.64	0.63	4.01	1.41	4.85	0.42	4.70	0.56	4.56	0.83	4.65	0.01	0.03
Self-efficacy—training activities	4.58	0.66	4.72	0.54	4.50	0.94	4.68	0.58	4.57	0.66	4.01	0.01	4.78	0.46	4.70	0.64	4.58	0.66	3.20	0.01	0.02
Self-efficacy—household tasks	4.51	0.88	4.66	0.69	4.43	0.76	4.52	0.75	4.56	0.75	5.01	0.01	4.79	0.52	4.74	0.62	4.51	0.88	3.09	0.01	0.02
Self-efficacy—personal care	4.84	0.37	4.91	0.35	4.79	0.43	4.86	0.46	4.82	0.52	4.50	0.71	4.94	0.29	4.91	0.42	4.84	0.37	3.31	0.01	0.02
Total self-efficacy	40.93	4.67	42.33	3.74	40.86	3.88	41.72	4.39	40.80	4.91	33.01	4.24	43.03	2.98	41.83	4.40	40.93	4.67	4.73	0.01	0.04

### Differential Patterns of Practices That Promote Quality of Life and Use of Web 2.0 Tools According to Favorable Life Experiences

Although, variability was found according to the type of tool, generally speaking, life experiences related to physical health, important life changes (e.g., birth of a child), finances and education appeared to be associated with greater use of web 2.0 tools (Table 3). Thus, for example, frequency of browser use seemed to be higher among subjects whose most favorable life events were related to physical health, major life changes, education and social life (e.g., USE of browsers, *M*_lifechange_ = 4.89 vs. *M*_job_ = 2.51, *p* = 0.01). With regard to the benefits of the use of web 2.0 tools, statistically significant results were obtained in relation to personal satisfaction. In particular, those who reported positive events related to finance, law, mental health, leisure and tourism, sports and spirituality, among others, seemed to experience higher levels of personal satisfaction derived from the use of these tools.

**Table 3:** Differential patterns in quality of life practices, use of web 2.0 tools, and perceived benefits according to favorable life experiences.

**Variables**	**Physical health**		**Mental health**		**Social**		**Finances**		**Job**		**Education**		**Legal**		**Important life change**		**Other**		***F***	***p***	**η^**2**^**
	**$\bar{\text{X}}$**	**σ**	**$\bar{\text{X}}$**	**σ**	**$\bar{\text{X}}$**	**σ**	**$\bar{\text{X}}$**	**σ**	**$\bar{\text{X}}$**	**σ**	**$\bar{\text{X}}$**	**σ**	**$\bar{\text{X}}$**	**σ**	**$\bar{\text{X}}$**	**σ**	**$\bar{\text{X}}$**	**σ**			
**FREQUENCY OF QUALITY OF LIFE PRACTICES**																					
Personal hygiene	4.91	0.34	5.01	0.01	4.90	0.41	4.83	0.45	5.01	0.01	4.96	0.24	5.01	0.01	4.91	0.34	5.01	0.01	1.97	0.05	0.02
Healthy eating	4.21	0.83	3.93	1.01	4.23	1.01	3.95	0.99	3.50	0.71	4.50	0.81	4.17	0.89	4.21	0.83	3.93	1.01	2.35	0.02	0.02
Self health care	4.38	0.76	4.14	0.66	4.37	0.80	4.10	0.90	3.50	0.71	4.54	0.67	4.04	1.07	4.38	0.76	4.14	0.66	3.05	0.01	0.02
Consumption of harmful substances	2.22	1.22	1.43	0.76	2.13	1.19	2.48	1.25	1.50	0.71	2.16	1.26	2.26	1.05	2.22	1.22	1.43	0.76	2.47	0.01	0.02
Taking daily decisions	4.64	0.62	4.43	0.65	4.68	0.65	4.51	0.67	4.01	1.41	4.78	0.50	4.74	0.45	4.64	0.62	4.43	0.65	2.84	0.01	0.02
Taking important decisions	4.59	0.70	4.64	0.63	4.63	0.71	4.48	0.75	3.50	0.71	4.75	0.56	4.52	0.67	4.59	0.70	4.64	0.63	2.26	0.02	0.02
Money management	4.70	0.67	4.43	0.85	4.65	0.79	4.50	0.78	4.01	0.01	4.75	0.73	4.61	0.66	4.70	0.67	4.43	0.85	2.33	0.02	0.02
Access to necessary material things	4.51	0.67	4.43	0.85	4.59	0.60	4.33	0.68	4.01	0.01	4.61	0.68	4.22	0.85	4.51	0.67	4.43	0.85	1.94	0.05	0.02
Own rights defended by other people	3.53	0.98	3.71	1.20	3.85	0.96	3.35	1.12	2.50	0.71	3.88	1.04	3.70	1.15	3.53	0.98	3.71	1.20	3.70	0.01	0.03
Physical activity	4.01	0.97	3.86	1.10	4.22	0.98	3.74	1.12	2.01	1.41	4.08	0.99	4.01	0.85	4.01	0.97	3.86	1.10	3.07	0.01	0.02
Visits from friends	2.98	0.92	2.43	0.85	2.69	0.92	3.12	1.05	4.01	0.01	2.94	0.89	3.01	1.13	2.98	0.92	2.43	0.85	3.68	0.01	0.03
Contact with friends	4.50	0.70	4.14	0.86	4.24	0.92	4.43	0.76	5.01	0.01	4.42	0.70	4.61	0.66	4.50	0.70	4.14	0.86	2.29	0.02	0.02
Intimate relationships	3.12	1.13	2.93	1.44	2.51	1.28	2.92	1.20	2.01	0.01	2.94	1.24	2.65	1.30	3.12	1.13	2.93	1.44	3.78	0.01	0.03
Household tasks	4.55	0.76	4.64	0.50	4.59	0.68	4.46	0.79	5.01	0.01	4.68	0.73	4.74	0.45	4.55	0.76	4.64	0.50	2.14	0.03	0.02
**USE OF WEB 2.0 TOOLS AND PERCEIVED BENEFITS**																					
Use of browsers	4.89	0.74	3.93	2.13	4.82	0.94	4.64	1.31	2.51	3.54	4.83	0.92	4.78	1.04	4.89	0.74	3.93	2.13	3.81	0.01	0.03
Use of office automation tools	4.49	1.51	3.57	2.34	4.04	1.97	4.27	1.77	2.51	3.54	4.55	1.43	4.13	1.94	4.49	1.51	3.57	2.34	3.22	0.01	0.02
Use of functional tools	4.51	1.48	2.50	2.59	3.97	2.03	4.32	1.72	2.51	3.54	4.18	1.85	4.13	1.94	4.51	1.48	2.50	2.59	3.96	0.01	0.03
Use of image and sound tools	4.07	1.95	2.86	2.57	3.05	2.45	4.01	2.01	2.51	3.54	3.86	2.10	3.91	2.11	4.07	1.95	2.86	2.57	3.94	0.01	0.03
Use of social networks	3.78	2.15	2.86	2.57	3.05	2.45	3.96	2.04	5.01	0.01	3.52	2.29	4.35	1.72	3.78	2.15	2.86	2.57	2.18	0.03	0.02
Use of communication tools	4.51	1.48	3.21	2.49	3.93	2.06	4.32	1.72	2.50	3.54	4.53	1.46	3.91	2.11	4.51	1.48	3.21	2.49	3.03	0.01	0.02
Use of cloud tools	3.39	2.34	1.43	2.34	2.54	2.51	3.33	2.37	2.51	3.54	3.17	2.42	2.83	2.53	3.39	2.34	1.43	2.34	2.81	0.01	0.02
Use of educational tools	2.69	2.50	1.79	2.49	1.80	2.41	3.23	2.40	2.51	3.54	2.85	2.48	2.83	2.53	2.69	2.50	1.79	2.49	2.48	0.01	0.02
Benefits-personal satisfaction	3.12	1.12	3.64	1.50	3.15	1.09	3.21	0.89	3.01	0.01	2.97	1.12	3.48	0.79	3.12	1.12	3.64	1.50	2.18	0.03	0.02

### Differential Patterns of Perceived Quality of Life and Psychological Profiles According to Stressful Life Experiences

As with favorable life experiences, stressful events were also associated with differential patterns in perceived quality of life and psychological profiles. Thus, for example, addiction was associated with lower levels of perceived quality of life, whereas the highest levels were associated with the category of other experiences, which included experiences of prison, war or armed conflict, physical and psychological abuse, life transitions (retirement, working life), and abortion (e.g., total perceived quality of life, *M*_addiction_ = 39.71 vs. *M*_other_ = 48.59, *p* = 0.01) (Table 4). Similarly, differential patterns depending on the type of stressful life experience in factors constituting the subjects' psychological profiles were found, including: emotional expression, size of social network, feelings of loneliness, and self-efficacy in relation to management of finances.

**Table 4:** Differential patterns in perceived quality of life and psychological profiles according to stressful life experiences.

**Variables**	**Death**		**Education**		**PI**		**MI**		**ACC**		**S/D**		**Job**		**Finances**		**Leaving home**		**Addiction**		**Other**		***F***	***p***	**η^**2**^**
	**$\bar{\text{X}}$**	**σ**	**$\bar{\text{X}}$**	**σ**	**$\bar{\text{X}}$**	**σ**	**$\bar{\text{X}}$**	**σ**	**$\bar{\text{X}}$**	**σ**	**$\bar{\text{X}}$**	**σ**	**$\bar{\text{X}}$**	**σ**	**$\bar{\text{X}}$**	**σ**	**$\bar{\text{X}}$**	**σ**	**$\bar{\text{X}}$**	**σ**	**$\bar{\text{X}}$**	**σ**			
**PERCEIVED QUALITY OF LIFE**																									
Family	3.56	0.56	3.33	0.71	3.25	0.62	2.87	1.06	3.08	0.76	2.93	0.63	3.07	0.62	3.50	0.58	3.56	0.51	2.88	0.84	3.43	3.56	0.56	3.33	0.71
Intimate partner	2.88	0.97	2.67	1.10	2.93	0.90	2.77	0.99	2.89	1.09	2.32	1.08	2.88	1.07	2.85	0.99	2.81	0.98	2.2	1.23	3.02	2.88	0.97	2.67	1.10
Money	2.88	0.62	2.58	0.84	2.95	0.63	2.72	0.86	2.78	0.70	2.78	0.76	2.38	0.78	2.30	0.73	2.68	0.76	2.01	0.94	2.92	2.88	0.62	2.58	0.84
Occupation	3.01	0.74	2.50	0.94	2.95	0.71	2.64	0.93	2.67	1.01	2.89	0.90	2.29	0.94	2.45	0.83	2.90	0.83	1.9	0.99	2.99	3.01	0.74	2.50	0.94
Personal satisfaction	3.20	0.61	2.92	0.73	3.13	0.51	2.82	0.64	3.01	0.73	3.01	0.63	2.88	0.81	3.20	0.52	3.07	0.72	2.3	0.48	3.25	3.20	0.61	2.92	0.73
Life in general	3.21	0.55	3.03	0.74	3.06	0.43	2.95	0.61	3.19	0.68	2.96	0.63	3.03	0.63	3.01	0.32	3.12	0.56	2.4	0.52	3.33	3.21	0.55	3.03	0.74
Total perceived quality of life	48.05	5.48	46.25	6.64	47.01	4.88	45.21	5.89	47.30	6.73	45.96	5.99	45.38	6.23	46.90	5.26	46.42	6.75	39.71	6.72	48.59	48.05	5.48	46.25	6.64
**PSYCHOLOGICAL PROFILE**																									
Emotional intelligence—emotional expression	3.58	0.98	3.01	0.86	3.74	0.84	3.46	1.02	3.44	1.22	3.41	1.03	3.65	0.81	3.9	0.91	3.27	1.21	3.4	1.51	3.02	1.01	2.03	0.01	0.03
Social dimension—size of social network	3.69	1.05	3.22	1.22	3.7	1.05	3.21	1.15	3.59	1.31	3.46	1.24	2.94	1.07	3.55	1.15	3.46	1.05	2.5	1.58	3.53	1.06	1.97	0.011	0.032
Social dimension—loneliness	3.89	1.09	3.42	1.16	4.09	0.97	3.62	1.21	4.19	0.88	3.44	1.22	3.65	1.13	3.85	0.99	3.78	1.06	3.4	1.51	3.68	1.13	2.7	0.001	0.043
Self-efficacy—management of finances	4.85	0.41	4.69	0.53	4.73	0.49	4.72	0.51	4.74	0.59	4.58	0.63	4.77	0.61	4.65	0.67	4.73	0.55	4.01	0.94	4.81	.68	1.89	0.02	0.03

*Death, Death of a loved one; PI, Physical illness; MI, Mental illness; ACC, Severe accident; S/D, Separation/divorce*.

### Differential Patterns of Practices That Promote Quality of Life and Use of Web 2.0 Tools According to Stressful Life Experiences

Differential patterns in practices that promote quality of life and the use of web 2.0 tools were also observed (Table 5). In general, the death of a loved one was associated with greater subsequent practice of activities that promote quality of life. However, there were exceptions. For example, the death of a loved one was also related to greater reliance on consumption of substances harmful to health (e.g., consumption of harmful substances, *M*_deathofalovedone_ = 1.77 vs. *M*_mentalillness_ = 2.8, *p* = 0.01). Regarding the use of web 2.0 tools, stressful life experiences were again associated with differential patterns. For example, subjects who reported educational problems tended to make more use of social networks (e.g., USE of social networks, *M*_education_ = 4.72 vs. *M*_addiction_ = 3.01, *p* = 0.01). Similarly, differences in relation to the perceived benefits arising from the use of web 2.0 tools were found. Thus, subjects who had suffered the death of a loved one perceived greater physical and mental health benefits (e.g., health BENEFITS, *M*_deathofalovedone_ = 3.19 vs. *M*_other_ = 2.19, *p* = 0.01).

**Table 5:** Differential patterns in quality of life practices, use of web 2.0 tools, and perceived benefits according to stressful life experiences.

**Variables**	**Death**		**Education**		**PI**		**MI**		**ACC**		**S/D**		**Job**		**Finances**		**Leaving home**		**Addiction**		**Other**		***F***	***p***	**η^**2**^**
	**$\bar{\text{X}}$**	**σ**	**$\bar{\text{X}}$**	**σ**	**$\bar{\text{X}}$**	**σ**	**$\bar{\text{X}}$**	**σ**	**$\bar{\text{X}}$**	**σ**	**$\bar{\text{X}}$**	**σ**	**$\bar{\text{X}}$**	**σ**	**$\bar{\text{X}}$**	**σ**	**$\bar{\text{X}}$**	**σ**	**$\bar{\text{X}}$**	**σ**	**$\bar{\text{X}}$**	**σ**			
**FREQUENCY OF QUALITY OF LIFE PRACTICES**																									
Self health care	4.63	0.60	3.92	0.84	4.41	0.67	4.33	0.84	4.26	0.86	4.36	0.80	4.50	0.71	4.35	0.88	4.39	0.70	3.70	0.95	4.31	0.84	3.03	0.01	0.05
Consumption of harmful substances	1.77	1.06	2.61	1.18	2.12	1.26	2.8	1.15	2.19	1.21	2.44	1.22	1.88	0.91	2.35	1.39	2.32	1.17	2.90	1.20	2.07	1.24	3.02	0.01	0.05
Taking daily decisions	4.79	0.48	4.44	0.70	4.67	0.53	4.59	0.60	4.59	0.64	4.72	0.53	4.50	0.93	4.70	0.66	4.59	0.63	4.80	0.42	4.62	0.67	1.66	0.05	0.03
Taking important decisions	4.76	0.58	4.31	0.86	4.68	0.61	4.51	0.68	4.48	0.70	4.66	0.63	4.53	0.83	4.65	0.67	4.61	0.59	4.90	0.32	4.76	0.75	1.92	0.01	0.03
Decisions taken by other people	4.19	0.86	3.81	0.82	4.11	0.88	3.77	0.81	4.11	0.80	4.19	0.76	4.24	0.78	3.95	0.89	3.83	1.02	4.01	0.94	4.26	0.83	1.63	0.05	0.03
Control of negative emotions	4.18	0.83	3.69	0.95	4.08	0.73	3.44	1.02	4.11	0.75	3.97	0.90	4.03	0.87	4.01	0.73	3.98	0.79	4.01	0.82	4.25	0.94	2.46	0.01	0.04
Money management	4.81	0.56	4.28	1.03	4.66	0.80	4.39	0.99	4.78	0.51	4.67	0.62	4.59	0.82	4.60	0.75	4.76	0.54	4.20	1.03	4.69	0.80	2.49	0.01	0.04
Access to necessary material things	4.69	0.56	4.22	0.76	4.62	0.59	4.39	0.67	4.41	0.84	4.48	0.66	4.27	0.75	4.35	0.93	4.44	0.59	3.80	1.14	4.57	0.71	3.86	0.01	0.06
Defense of own rights	4.53	0.70	3.83	1.08	4.37	0.75	4.26	0.64	4.37	0.79	4.32	0.85	4.27	0.71	4.50	0.69	4.12	0.90	4.50	0.85	4.28	0.80	1.75	0.03	0.03
Own rights defended by other people	3.90	1.05	3.11	1.06	3.61	1.01	3.41	0.85	3.74	0.98	3.59	1.05	3.97	1.01	3.70	0.98	3.34	0.99	3.10	0.99	3.84	1.03	2.58	0.01	0.04
Legal help	2.99	1.32	2.86	1.25	2.71	1.27	2.74	1.31	2.37	1.31	2.58	1.13	2.59	1.10	2.70	1.30	2.34	1.30	3.01	1.49	2.74	1.13	2.04	0.01	0.03
Physical activity	4.23	1.07	3.50	1.03	3.99	0.93	3.95	0.95	4.15	1.06	4.17	0.92	4.15	0.96	3.85	0.75	4.01	0.98	3.30	1.42	3.90	1.10	2.24	0.01	0.04
Mental activity	4.64	0.82	4.22	0.99	4.27	0.99	4.08	1.13	4.37	0.84	4.40	1.03	4.38	0.82	4.30	0.80	4.34	1.02	3.50	1.78	4.21	0.99	2.34	0.01	0.04
Visits from family	3.61	0.95	3.28	1.34	3.47	0.96	3.08	1.01	3.19	1.21	3.06	1.06	3.21	1.01	3.40	0.88	3.20	1.15	3.30	0.82	3.25	1.04	1.66	0.05	0.03
Visits from friends	2.99	0.91	3.31	0.95	2.96	0.93	2.74	1.04	2.67	0.92	2.87	0.97	2.59	0.66	2.95	0.61	3.20	0.96	2.80	1.14	3.40	0.98	1.94	0.01	0.03
Family reunion outside the home	3.62	0.85	2.97	0.94	3.47	0.82	3.23	1.04	3.26	0.86	3.30	0.89	3.06	0.98	3.50	0.83	3.29	0.98	2.60	0.97	3.07	0.90	2.32	0.01	0.04
Contact with family	4.55	0.65	4.22	1.12	4.51	0.59	4.23	0.90	4.15	1.03	4.28	0.94	4.18	1.01	4.35	0.75	4.22	0.91	3.80	0.92	4.35	0.81	1.73	0.03	0.03
Intimate relationships	2.79	1.08	3.22	1.07	3.27	1.11	3.13	1.11	2.70	1.10	2.64	1.28	2.94	1.30	2.85	1.18	3.05	1.32	3.10	1.29	2.79	1.21	4.31	0.01	0.07
Household tasks	4.57	0.91	4.56	0.65	4.58	0.76	4.36	0.81	4.63	0.63	4.55	0.75	4.47	0.79	4.55	0.76	4.63	0.62	4.20	1.23	4.53	0.75	1.70	0.04	0.03
Leisure at home	4.65	0.69	4.44	0.65	4.31	0.98	4.44	0.91	4.19	0.79	4.53	0.71	4.29	0.84	4.35	0.67	4.66	0.66	4.50	0.97	4.43	0.81	2.54	0.01	0.04
**USE OF WEB 2.0 TOOLS AND PERCEIVED BENEFITS**																									
Use of image and sound tools	3.53	2.23	4.86	0.83	3.83	2.13	4.36	1.69	4.26	1.81	4.14	1.90	3.68	2.24	4.01	2.05	4.27	1.79	4.50	1.58	4.02	1.95	2.22	0.01	0.04
Use of social networks	3.27	2.42	4.72	1.16	3.42	2.33	3.85	2.13	3.52	2.33	4.14	1.90	3.97	2.05	4.25	1.83	4.51	1.50	3.01	2.58	3.64	2.06	2.71	0.01	0.04
Use of cloud tools	2.52	2.51	4.31	1.75	3.33	2.37	3.46	2.34	2.22	2.53	3.50	2.30	3.09	2.47	4.01	2.05	4.02	2.01	2.50	2.64	2.99	2.32	2.72	0.01	0.04
Use of tools to select, organize, and share information	0.99	1.98	3.19	2.44	1.35	2.23	1.67	2.39	1.11	2.12	1.23	2.16	1.50	2.35	1.34	2.24	1.01	2.11	2.50	3.54	1.31	2.23	2.42	0.01	0.04
Use of educational tools	1.68	2.37	4.58	1.40	2.75	2.50	3.21	2.43	2.96	2.50	2.41	2.51	2.21	2.52	3.01	2.51	3.42	2.36	2.50	2.64	4.17	2.29	5.26	0.01	0.08
Benefits—physical and mental health	3.19	1.15	2.78	1.07	2.99	1.06	3.03	0.90	3.15	1.17	2.86	0.94	2.85	1.21	2.65	0.88	3.05	1.12	3.01	0.94	2.19	1.01	2.16	0.01	0.04
Benefits—personal satisfaction	3.34	1.17	2.92	1.23	3.21	0.99	2.87	1.11	3.63	1.12	2.96	1.11	3.15	1.11	2.65	0.99	3.10	1.02	3.10	0.88	2.91	1.04	1.72	0.03	0.03

*Death, Death of a loved one; PI, Physical illness; MI, Mental illness; ACC, Severe accident; S/D, Separation/divorce*.

## Discussion

The study objective was achieved, namely to answer the initial research question. Both favorable and stressful life experiences of adults and older adults were associated differential patterns in intimate and personal aspects such as psychological profiles and perceived quality of life, and in daily activities and practices that influence quality of life, such as the use of web 2.0 tools, which yielded cognitive, affective, social, emotional, physical, and behavioral benefits, among others (Chen and Schulz, 2016; Díaz-Prieto and García-Sánchez, 2016; Khosravi and Ghapanchi, 2016). In spite of this, differences according to age and gender were not found.

One of the main contributions of this study is the analysis of the role of favorable life experiences. To date, most studies have focused on stressful life events and their consequences (Kendler and Gardner, 2016; Mayo et al., 2017; Pan et al., 2017). However, our study demonstrates that favorable life experiences also play an important role, mainly in the construction of an individual's psychological profile and use of web 2.0 tools, but also in perceived quality of life and engagement in practices that promote said quality. Favorable life experiences were associated with a higher number of differences in the subjects' psychological profiles and use of web 2.0 tools, whereas stressful life experiences were associated with greater differential patterns in perceived quality of life and quality of life practices. It is the combined experiences of an adult or older adult that determine his or her identity, personality and lifestyle and affect his or her physical and mental health status and well-being (Blonski et al., 2016).

Traditionally, past adverse or stressful life events have been associated with a life marked by negative events (Lim and DeSteno, 2016). In line with more recent models and theories such as adaptation to development and resilience (Cho et al., 2015), our study demonstrates that stressful life experiences sometimes act as powerful catalysts, spurring individuals to overcome these events and triggering a process of personal growth that exerts a positive effect psychologically and with regard to daily practices and activities.

In relation to perceived quality of life, life experiences were associated with differences in terms of interpersonal relationships, finances, occupation, satisfaction, assessment of life in general, and the global quality of life score, with differential patterns according to whether the experience was favorable or stressful and the specific type of experience, although no clear trend was observed. Several studies have previously reported the impact of life experiences on quality of life and well-being (Pocnet et al., 2017), but few have described the specific areas influenced.

As regards psychological profiles, favorable and stressful life experiences alike shaped the emotional, social, and self-efficacy dimensions, but only favorable experiences were associated with differences in motivation. Whereas, previous studies have reported the impact of mainly stressful life experiences on psychological profiles (Lasgaard et al., 2016), our study shows that favorable experiences also contribute to their construction.

In terms of the frequency of engaging in empirical evidence-based practices that promote quality of life, favorable, and stressful life experiences alike were associated with differential patterns in physical and mental health, self-determination, social, material, and functional domains and emotional well-being. All this contributes to enhancing the quality of life, as evidenced by various studies (Gómez et al., 2015).

Lastly, with regard to the use of web 2.0 tools and the perceived benefits of this, life experiences, in this case mainly favorable ones, were associated with differential patterns of use for almost all of the tools studied. Nevertheless, unfavorable life experiences were associated with greater differences in the perceived benefits in relation to physical and mental health and personal satisfaction. This may be because better standards of living probably favor the use of these media. In fact, several previous studies have confirmed the relationship between a higher socioeconomic level and greater use of the internet and web 2.0 tools in various age groups (Lai and Kwan, 2017). Our results in relation to the benefits indicate that these tools possess significant potential as a coping resource, in agreement with previous studies (Li et al., 2016).

Nonetheless, despite these differential patterns, the differences between them were very small and no clear trends were observed. This may be because emotional associations may exert a greater influence regardless of the type of experience. It might also be explained in light of theories such as resilience, according to which, people tend to focus on positive events and to deploy a series of strategies for coping with stressful events in order to promote positive adaptation (Randall et al., 2015).

This study presents a series of limitations that must be taken into consideration. First, use of the tool *Google Forms* entailed problems related to privacy, the impossibility of establishing a password, technical problems that made it necessary to eliminate responses and the impossibility of saving responses when answering the questionnaire, which could lead to loss of all information entered in the event of a connection or application failure. In general, the researchers were able to overcome these difficulties, making the necessary adjustments during the design process and questionnaire administration alike, and thus they did not affect the results to any great extent. However, many of these difficulties could be avoided by administering the questionnaire via another type of tool such as *SurveyMonkey*. It is also necessary to note the possible existence of sample bias. For example, since voluntary sampling was used, factors such as participant motivation to complete the questionnaire, the availability of technological resources or the need for basic digital competence may have influenced the final sample obtained. Different results to ours might be obtained in populations with other sociodemographic, economic, and educational characteristics, and it is therefore difficult to generalize our findings. In addition, people with problems of autonomy were not included, and this would be an interesting future avenue to explore using another type of instrument through individualized questionnaires. Furthermore, although this study included a representative sample of adults and older adults from various parts of Spain, it may nevertheless be necessary to conduct a comparative study of the different regions in Spain which would complement the results obtained here.

This study underscores the impact of favorable and stressful life experiences on perceived quality of life, psychological profiles, and practices that promote quality of life, including the use of web 2.0 tools. Differential patterns in relation to life experiences were found, indicating that besides considering sociodemographic factors, as has been the custom to date, interventions aimed at improving quality of life should also take into account life experiences and the other factors analyzed here. In this way, this study could have important implications at a theoretical, practical, political, psychological, educational, and social level. On the one hand, because the findings obtained contribute to enrich knowledge in relation to the study of life experiences in adulthood and in old age. On the other hand, because this could contribute to the implementation of more successful psychological and social interventions within the framework of Educational and Social Gerontology. Finally, because this study opens the doors to education in life experiences, education for coping with life problems, addressing its implications in the mentioned psychoeducational constructs, as well as for the promotion of development at different ages and throughout the life cycle. This could contribute to the implementation of more successful psychological and social interventions and attract the attention of social policies.

## Data Availability Statement

The datasets generated for this study are available on request to the corresponding author.

## Ethics Statement

This study was reviewed, approved, and carried out in accordance with the recommendations of the scientific and research Ethical Committee of Universidad de León, with written informed consent from participants through the App used, before all subjects were enrolled in our study.

## Author Contributions

CD-P and J-NG-S designed the study and wrote the article. CD-P reviewed the antecedents and implemented the study. J-NG-S performed the data analysis. AC-G reviewed and contributed instrumentally in the study as a whole and its implementation. All authors made relevant contributions in the final result.

### Conflict of Interest

The authors declare that the research was conducted in the absence of any commercial or financial relationships that could be construed as a potential conflict of interest.
